# Coral bleaching resistance variation is linked to differential mortality and skeletal growth during recovery

**DOI:** 10.1111/eva.13500

**Published:** 2022-11-07

**Authors:** Nia S. Walker, Victor Nestor, Yimnang Golbuu, Stephen R. Palumbi

**Affiliations:** ^1^ Department of Biology Hopkins Marine Station of Stanford University Pacific Grove California USA; ^2^ Hawaiʻi Institute of Marine Biology University of Hawai‘i at Mānoa Kāneʻohe Hawaii USA; ^3^ Palau International Coral Reef Center Koror Palau

**Keywords:** *Acropora hyacinthus*, heat stress resistance and recovery, intraspecific variation, thermal resilience, tradeoffs

## Abstract

The prevalence of global coral bleaching has focused much attention on the possibility of interventions to increase heat resistance. However, if high heat resistance is linked to fitness tradeoffs that may disadvantage corals in other areas, then a more holistic view of heat resilience may be beneficial. In particular, overall resilience of a species to heat stress is likely to be the product of both resistance to heat and recovery from heat stress. Here, we investigate heat resistance and recovery among individual *Acropora hyacinthus* colonies in Palau. We divided corals into low, moderate, and high heat resistance categories based on the number of days (4–9) needed to reach significant pigmentation loss due to experimental heat stress. Afterward, we deployed corals back onto a reef in a common garden 6‐month recovery experiment that monitored chlorophyll *a*, mortality, and skeletal growth. Heat resistance was negatively correlated with mortality during early recovery (0–1 month) but not late recovery (4–6 months), and chlorophyll *a* concentration recovered in heat‐stressed corals by 1‐month postbleaching. However, moderate‐resistance corals had significantly greater skeletal growth than high‐resistance corals by 4 months of recovery. High‐ and low‐resistance corals on average did not exhibit skeletal growth within the observed recovery period. These data suggest complex tradeoffs may exist between coral heat resistance and recovery and highlight the importance of incorporating multiple aspects of resilience into future reef management programs.

## 1 INTRODUCTION

A significant number of interventions to increase coral reef resilience involve finding, protecting, breeding, or propagating heat‐resistant corals (NAS, 2019). However, interventions based on heat resistance also have associated risks. First, it is likely that heat resistance is not the only driver of future coral reef health (Kornder et al., 2018), and selection for heat‐resistant colonies is not guaranteed to enhance other attributes needed in the future, such as disease susceptibility, growth, or reproduction (Muller et al., 2018, 2020; NAS, 2019). Second, evolutionary theory predicts that genes in a population leading to coral heat resistance are likely to confer disadvantages as well; otherwise, all corals would have these high‐resistant genotypes (Calosi et al., 2016). As a result, there may be negative correlations between heat resistance and other features of coral fitness. For example, tradeoffs between heat resistance and growth have been previously reported (Cornwell et al., 2021; Mieog et al., 2009), though this can be habitat (Bay & Palumbi, 2017) or species‐dependent (Morikawa & Palumbi, 2019). Third, heat resistance is just one aspect of coral resilience, especially as heat stress‐induced bleaching is expected to increase (Hoegh‐Guldberg et al., 2007; Hughes et al., 2003, 2017, 2018; Pandolfi et al., 2011), and high heat‐resistant corals currently residing in hotter reef environments are predicted to exhibit differences in heat tolerance and resilience in future warmer climates (Voolstra et al., 2021).

The concept of resilience in the face of environmental stress has been linked to two critical features: resistance and recovery (Levin & Lubchenco, 2008). Resistance is defined as the ability of ecosystems, populations, or individuals to withstand negative impacts of extrinsic disturbances, whereas recovery is the ability to rebound after sustaining damage due to stress (Levin & Lubchenco, 2008). When applied to coral stress resilience, this suggests that there are primary roles for both resistance and recovery differences in establishing the thermal resilience of individual corals. The functional importance of resistance and recovery suggests that their correlation is also fundamental to resilience dynamics. There is high intra‐ and interspecific variation in coral heat stress resistance (e.g., Cornwell et al., 2021; Morikawa & Palumbi, 2019; Schoepf et al., 2019; Thomas et al., 2018; Ulstrup et al., 2006) and recovery (e.g., Matsuda et al., 2020; Rodrigues & Grottoli, 2007; Schoepf, Grottoli, et al., 2015; Schoepf, Stat, et al., 2015; Walker et al., 2022), which allows for assays that can harness variation to evaluate heat stress resistance and recovery in tandem. Further, the coral holobiont transcriptome responds rapidly and significantly to heat stress (Savary et al., 2021; Seneca & Palumbi, 2015; Traylor‐Knowles et al., 2017), and correlates of faster recovery differ from transcriptome correlates of higher resistance (Thomas & Palumbi, 2017; Thomas et al., 2019), suggesting that components of the underlying genetic mechanisms of resistance and recovery differ. Mechanistically, higher endosymbiont chlorophyll, host energy reserves (i.e., lipids, carbohydrates, and proteins), and symbiont population growth rates decrease recovery times (Grottoli et al., 2017; Levas et al., 2013, 2018; Rodrigues & Grottoli, 2007; Schoepf, Grottoli, et al., 2015; Schoepf, Stat, et al., 2015). These basic features of coral holobiont metabolism and the coral‐symbiont mutualistic relationship may also increase resistance, generating positive correlations (Grottoli et al., 2014; Huffmyer et al., 2021; Schoepf, Grottoli, et al., 2015; Schoepf, Stat, et al., 2015; Thornhill et al., 2011).

In corals, comparisons of heat resistance and recovery suggest both positive and negative relationships between and within species. Faster recovery and lower mortality have been observed in thermally tolerant compared to thermally sensitive coral species following a natural heating event (Matsuda et al., 2020; Thomas et al., 2019). Other findings have suggested a negative relationship between species' skeletal growth rates and thermal tolerance (Carpenter et al., 2008; Rodrigues & Grottoli, 2007). Previously, it was found that 5 months after a natural bleaching event, *Acropora hyacinthus* high heat‐resistant individuals (i.e., no visual signs of bleaching) had higher energy reserves, a higher likelihood of containing gametes and higher amounts of oocytes per polyp compared to low heat‐resistant individuals (i.e., visible bleaching) who had visibly recovered (Leinbach et al., 2021). However, it remains largely unclear how heat resistance capacity is linked to heat recovery, or to fitness traits such as skeletal growth and reproduction.

Few studies to date have explicitly compared resistance and recovery among a set of coral colonies within a species (Leinbach et al., 2021; Matsuda et al., 2020; Morikawa & Palumbi, 2019; Walker et al., 2022). A consistent and important limitation of these comparisons is that corals with different levels of heat stress resistance experience the same amount of heat exposure, meaning that recovery is monitored in corals that sustained different levels of heat stress and bleaching. For example, highly heat‐resistant *A. hyacinthus* corals with little to no bleaching were shown to have significantly less mortality during the first 2 months of recovery compared to severely bleached low‐resistant corals after a standardized short‐term heat stress assay (Walker et al., 2022). In these experiments, like many others, there are two variables among colonies—the level of heat resistance and the level of bleaching—making it difficult to assess what is driving differences in recovery traits. As a result, it remains poorly understood whether high heat‐resistant corals can effectively recover when they eventually bleach, and if low heat‐resistant corals can recover well after bleaching. Therefore, it is important to evaluate recovery in corals with varying levels of heat stress resistance, in which individuals experienced different amounts of heat exposure time to achieve a similar degree of bleaching.

Here, we present the first study to investigate links between heat stress resistance and recovery in corals that sustained similar levels of bleaching. We subjected 39 tabletop corals (*A. hyacinthus*) to repeated, short‐term heat stress (1–9 days) until moderate bleaching was observed. We recorded relative bleaching resistance as the number of days to bleach. After corals were comparably bleached, we transplanted ramets from the 27 surviving coral colonies back onto their native reef environment in a common garden setting and tracked chlorophyll *a* content, mortality, and skeletal growth in a 6‐month recovery experiment. Our results show that genets with different levels of heat resistance restored chlorophyll *a* content within 1 month of recovery, and that heat resistance and mortality are negatively correlated. However, high‐ and low‐resistant corals had much slower poststress skeletal growth rates than did moderately resistant corals. Therefore, we provide evidence to suggest that the highest heat stress‐resistant corals might not exhibit the highest heat stress recovery when bleaching is induced.

## 2 MATERIALS AND METHODS

### 2.1 Site description and sample collection

**FIGURE 1:**
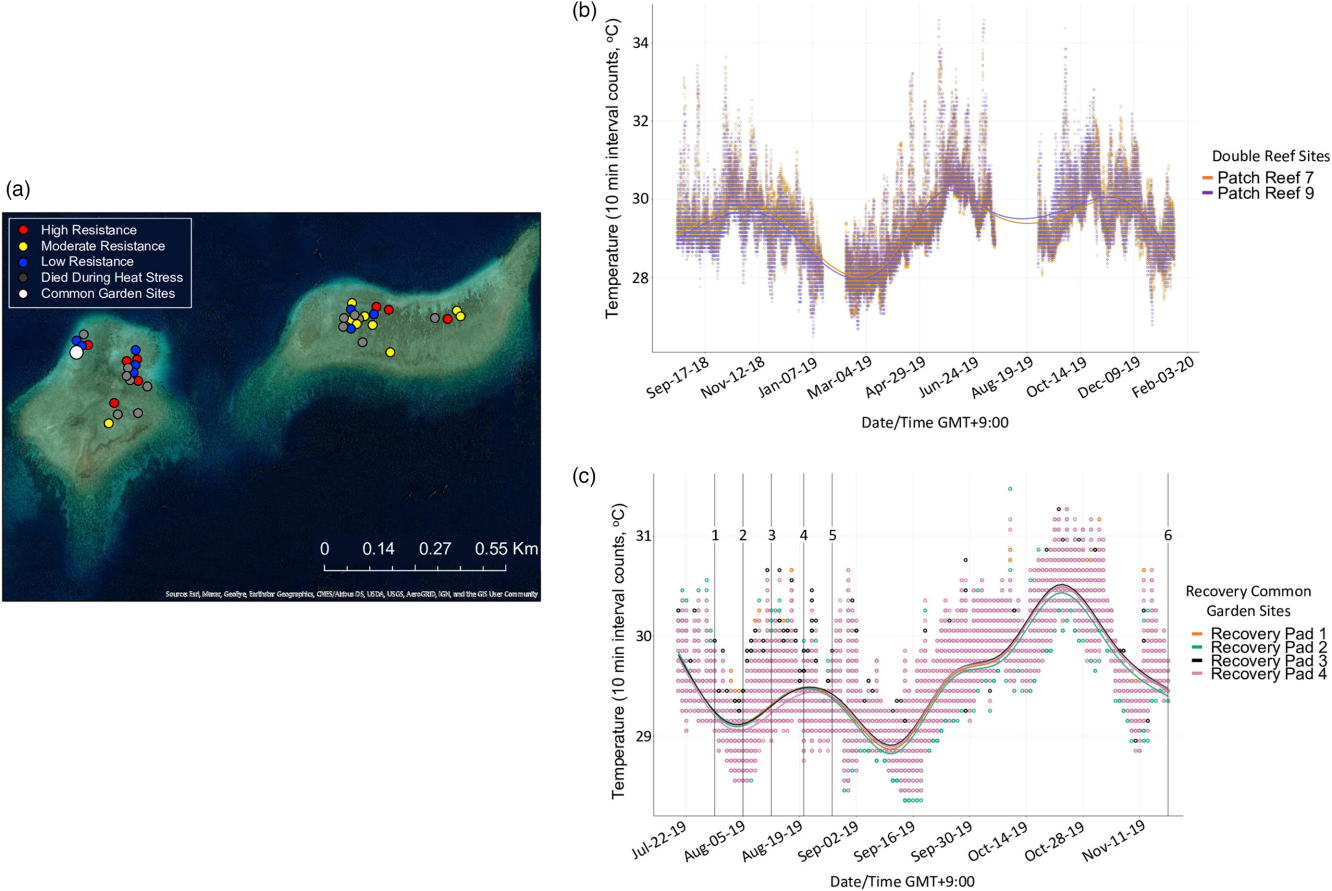
Geographic distribution of all coral colonies and temperature data. (a) ArcGIS‐generated map of double reef, located in the southern lagoon of Palau. The two patch reef sections were labeled patch reef 7 (on the righthand side, approximately N 7.29190 E 134.51034) and patch reef 9 (on the left, approximately N 7.29045 E 134.50325) in a previous widespread heat resistance mapping study (Cornwell et al., 2021). All corals were located within 1.5 km of each other, and each heat resistance category (high, moderate, and low) was represented on each patch reef, as well as corals that died during the heat stress experiment. The 6‐month recovery common garden experiment took place on patch reef 9 (N 7.29106, E 134.50215). (b) Timeseries plot of patch reef 7 and 9 temperature data 1 year prior to colony collection and during the recovery experiment period (August 19, 2018–January 20, 2020). We did not have available reef temperature data on January 19–February 10, 2019, July 18–August 30, 2019, or January 21–29 2020. (c) Timeseries plot of the 4 common garden concrete pads during 4 months of the recovery experiment (July 20, 2018–November 18, 2019). The plot includes vertical lines to highlight data collection timepoints: 1, recovery Day 0 (i.e., common garden deployment); 2, recovery Week 1; 3, recovery Week 2; 4, recovery Week 3; 5, recovery Month 1; 6, recovery Month 4. Temperature data at the common garden sites were unavailable during months 4–6 of the recovery period (November 19, 2019–January 29, 2020).

**FIGURE 2:**
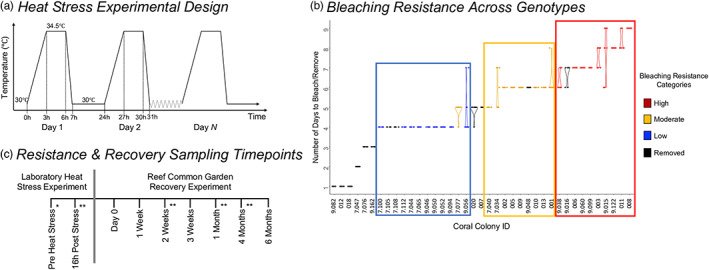
Heat stress resistance and recovery experiment design. (a) Daily heat stress ramp protocol. Heated ramets in the heat stress experiment ramped from 30 to 34.5°C then back down to 30°C daily, and controls were maintained at 30°C. Heated ramets remained in the heat stress experiment daily ramp cycle for however many days necessary to reach moderate bleaching (see Section 2; described in Walker et al., 2022). (b) Violin chart of days to reach target moderate bleaching across all genets, based on average number of days ramets bleached. Genets were divided into 3 equal broad heat resistance categories (high, moderate, low), based on average number of days ramets needed to bleach. Genets with excessive control and/or heated ramet mortality were also removed during the heat stress experiment. Coral Colony IDs (*x*‐axis) correspond to unique tags that were used to distinguish genets in the field and during the experiments. Colonies that begin with “7.” or labeled 002‐010 came from patch reef 7, while others with “9.” or labeled 011‐020 came from patch reef 9 (Cornwell et al., 2021; Walker et al., 2022). (c) Simplified timeline of sampling timepoints during the heat stress resistance and recovery experiments. The heat stress experiment was conducted in the laboratory, and then ramets were deployed onto a reef common garden environment for the recovery experiment. Ramet mortality was recorded at all timepoints, chlorophyll *a* and buoyant weight were measured at select poststress timepoints (**), and chlorophyll *a* was additionally measured prestress (*).

We collected branches from 39 *A. hyacinthus* colonies located on two sections of Double Reef in the Indo‐Pacific archipelago of Palau's southern lagoon (Patch Reefs 7 and 9 in Cornwell et al., 2021; Walker et al., 2022). All colonies were within 1.5 km of each other (Figure 1a), and we confirmed no colonies were identical using single nucleotide polymorphism genotypes called from transcriptome data (BioProject accession number PRJNA872206 in the NCBI BioProject database https://www.ncbi.nlm.nih.gov/bioproject/). Collection took place on July 18, 2019 and the fragments were bubble‐wrapped (Delbeek, 2008) and then transported via a 15 min boat ride to the Palau International Coral Reef Center (PICRC) for holding and heat stress experimentation. Upon returning to PICRC, fragments were placed into a 760 L holding tank outfitted with pumps (water turnover 1 full volume h^−1^) that received natural sunlight and ambient temperature (28–30°C) seawater from the surrounding southern lagoon. These fragments were clipped into 20 ramets per colony genet (~8 cm from base to the tallest branch)—10 control and 10 heated ramets (Figure 2). Twenty out of 39 coral colony genets were randomly chosen the following day to begin the acute heat stress experiment, due to space limitation in heat stress tanks. As initial genets were removed due to mortality or bleaching, the remaining 19 genets were added to the heat stress tanks as space allowed (3 colonies on 7/20/19 and 16 colonies on 7/21/19, further details in Appendix S1).

### 2.2 Heat stress experiment

There were two heated and two control tanks, each ~250 L. The tank setup consisted of heaters (100 and 300 W), a submersible pump (~280 L h^−1^), and a temperature controller (Inkbird ITC‐308). The heat stress experiment ran continuously over 11 days using a cyclical daily heat ramp system. The daily heat treatments ramped from 30 to 34.5°C over 3 h (10:00 AM–1:00 PM), held at 34.5°C for 3 h (1:00 PM–4:00 PM), and then ramped down over 1 h to 30°C and were held until the following day to repeat the ramp (Figure 2). The control tank setup was identical except water temperature was maintained at 30°C.

We targeted moderate bleaching in the heat stress experiment. Each morning prior to the heat ramp, two observers assessed all ramets for bleaching using a visual bleaching score metric: (1) no bleaching, (2) visible but minimal bleaching, (3) moderate bleaching, (4) severe bleaching, and (5) total bleaching (Cornwell et al., 2021; Walker et al., 2022). The visual bleaching score was used to initially determine when colonies were sufficiently bleached to remove from the heat stress experiment. Visual assessment of moderate bleaching was corroborated with chlorophyll *a* concentration, in which one control and heat‐stressed ramet per genet were sacrificed following removal (see Figure 4 and Appendix S1). We evaluated relative heat resistance based on the number of days to bleach. We also created three broad categories of relative heat resistance from this data: low, moderate, and high resistance (Figure 2, and see Appendix S1, also described in the first results section). Individual ramets were removed after moderate bleaching. In cases when intra‐colony ramets were removed on different days, we determined genet heat resistance based on average number of days to bleach (Figure 2). We also monitored mortality based on complete absence of coral tissue on the skeleton. Genets were removed from the heat stress experiment and discarded if 5 or more heated and/or control ramets died.

We also note that mortality of all control samples occurred in one heat stress experiment control tank on July 27, 2019 (only five genets remained), possibly due to an unknown disease or other issue in the one isolated tank. The two heated tanks and other control tank were not affected. We re‐collected ramets from those same colonies from the field (see Appendix S1) and deployed them onto the reef common garden. These control samples were not buoyantly weighed before deployment.

### 2.3 Common garden deployment and recovery experiment

Following the heat stress experiment, bleached and control ramets were returned to the large ambient temperature holding tank. Ramets were epoxied at the base to a plastic bolt and secured to plastic egg crates, so that one ramet per colony genet was represented on each egg crate. Controls and bleached ramets were placed on separate egg crates. Recovery panel egg crates were deployed onto Double Reef (Patch Reef 9, N 7.29106, E 134.50215) on July 29, 2019. Four egg crates each were cable tied to ~45 kg concrete pads, totaling 16 egg crates on 4 concrete pads separated by approximately 10–20 m. Temperature at all concrete pads was recorded at 10 min intervals from July 20 to November 18, 2019 (HOBO, OnSet Computing). We also utilized available average temperature data for Double Reef August 19, 2018 to January 20, 2020 (Palumbi, 2021). Temperature data show that 99.4% of all recorded counts were below 32°C, and there were no severe heating events in the year prior to the heat stress experiment (Figure 1b). Similarly, the recovery experiment thermal environment did not exceed 32°C (Figure 1c). The common garden environment was chosen to limit environmental variation during stress recovery. The recovery experiment spanned 6 months following common garden deployment. We monitored heated and control ramet total mortality on all egg crates at the following common garden timepoints: Day 0 (7/29), weekly for 1–4 weeks (8/5, 8/12, 8/20, 8/27), 4 months (11/18), and 6 months (1/29/20).

### 2.4 Physiological analyses

One control and heat‐stressed ramet per genet were randomly removed for destructive sampling (buoyant weight and chlorophyll *a*) at the following timepoints: preheat stress (just Chl *a*), 16 h postheat stress (just Chl *a*), and 2 weeks, 1 month, and 4 months after recovery common garden deployment (Figure 2). All ramets were nondestructively buoyantly weighed after the heat stress experiment but prior to common garden deployment for baseline skeletal weight (Jokiel et al., 1978). The percent change between buoyant weight at the recovery sampling timepoint and immediately postheat stress represented net calcification. Chlorophyll *a* was extracted at Hopkins Marine Station, USA from a clipped coral branch tip (~5 cm length) preserved in RNAlater. Samples were submerged in a vial with 4 ml of 95% EtOH for 10 min in darkness and centrifuged for 20 min at 4000 rpm. Spectrophotometric analyses of chlorophyll *a* were conducted using a universal quadrichroic equation (Ritchie, 2008). Chlorophyll *a* content was measured as heated divided by control ramet Chl *a* concentration and standardized to ramet surface area. Surface area was calculated in ImageJ (Schneider et al., 2012) by using cylindrical surface area (SA = 2πrh + 2πr^2^) as a proxy for individual branches within a ramet. It has been previously demonstrated that software such as ImageJ can be used for accurate surface area measurements on organisms with complex and irregular shapes, by breaking down parts into geometric figures for analysis (El‐Khaled et al., 2022; McLachlan et al., 2022; McLachlan & Grottoli, 2021). We further evaluated this metric by comparing ImageJ surface area values versus wax dipping (Veal et al., 2010), in an independent set of 50 *A. hyacinthus* coral fragments. The two methods were highly correlated (linear regression, *p* = 7.808 e^−16^, adjusted *R* ^2^ = 0.7446, Figure S1) and confirmed the validity of ImageJ‐generated surface area values.

### 2.5 Statistical analyses

All analyses were performed while using R software v4.2.0. Analyses were performed on mortality, chlorophyll, and skeletal weight data. We performed binomial linear mixed effects models to determine whether there was a significant relationship between mortality throughout the recovery period and genet heat resistance—based on number of days to bleach. Linear mixed effects models were generated for each postheat stress timepoint (16 h postheat stress, and then Day 0, Week 1, Week 2, Week 3, Month 1, Month 4, and Month 6 common garden recovery timepoints) with reef location, common garden concrete pad position, genotype, and control mortality included as random effects (lme4 package, v1.1‐29 in R); marginal *R* ^2^ values were also included for all models (sjstats package, v0.18.1). We also generated a mixed effects Cox proportional hazards model to determine likelihood of ramet survival throughout the recovery period depending on heat resistance category (low, moderate, or high) and genotype, including control survival as a random effect (coxme package, v2.2‐17 in R). Chlorophyll *a* concentration differences during pre‐ and post‐(~16 h post, then recovery Week 2, Month 1, and Month 4) heat stress timepoints were evaluated using Kruskal–Wallis and post hoc pairwise Wilcox tests (*p* < 0.05, fdr). To compare skeletal weight change throughout recovery (~16 h poststress, recovery Week 2, Month 1, and Month 4), we again performed Kruskal–Wallis and post hoc pairwise Wilcox tests (*p* < 0.05, fdr).

## 3 RESULTS

### 3.1 Intra‐colony consistency of the heat stress response

We subjected 39 coral genets to daily, short‐term heat stress until they experienced moderate bleaching; there were nine control and nine heated ramets per genet (Figure 2). Twenty‐seven colonies had most control and heated ramets (i.e., ≥5 out of 9 each) survive the heat stress experiment. Nineteen out of these 27 genets had 8–9 heat‐stressed ramets that reached the bleaching target on the same day. The other eight genets had 5–7 replicates that bleached within 1–2 days of one another (Figure 2b). Other replicates most often died (50%) or severely bleached (40%). These 27 genets were divided into three broad resistance groups based on the number of days to bleach, which allowed for comparing larger groups of genets with relatively different levels of heat resistance. Genets in the low‐resistance group bleached after four consecutive days (*n* = 9 genets), moderate‐resistance genets bleached after 5–6 days (*n* = 9 genets), and the high‐resistance group bleached after 7–9 days (*n* = 9 genets) (Figure 2b). Twelve of the assayed 39 genets rapidly experienced total mortality in all heated and/or control ramets. These genets were not included in further analyses in the heat resistance and recovery experiment. Ten out of 12 removed genets experienced total mortality of all ramets on the same day (Figure 2b). Overall, this short‐term heat stress system was able to reliably reveal intraspecific variation in heat resistance among colonies, whereby ramets within colonies consistently bleached or died due to heat stress.

### 3.2 Variation in mortality after heat stress based on heat resistance

**FIGURE 3:**
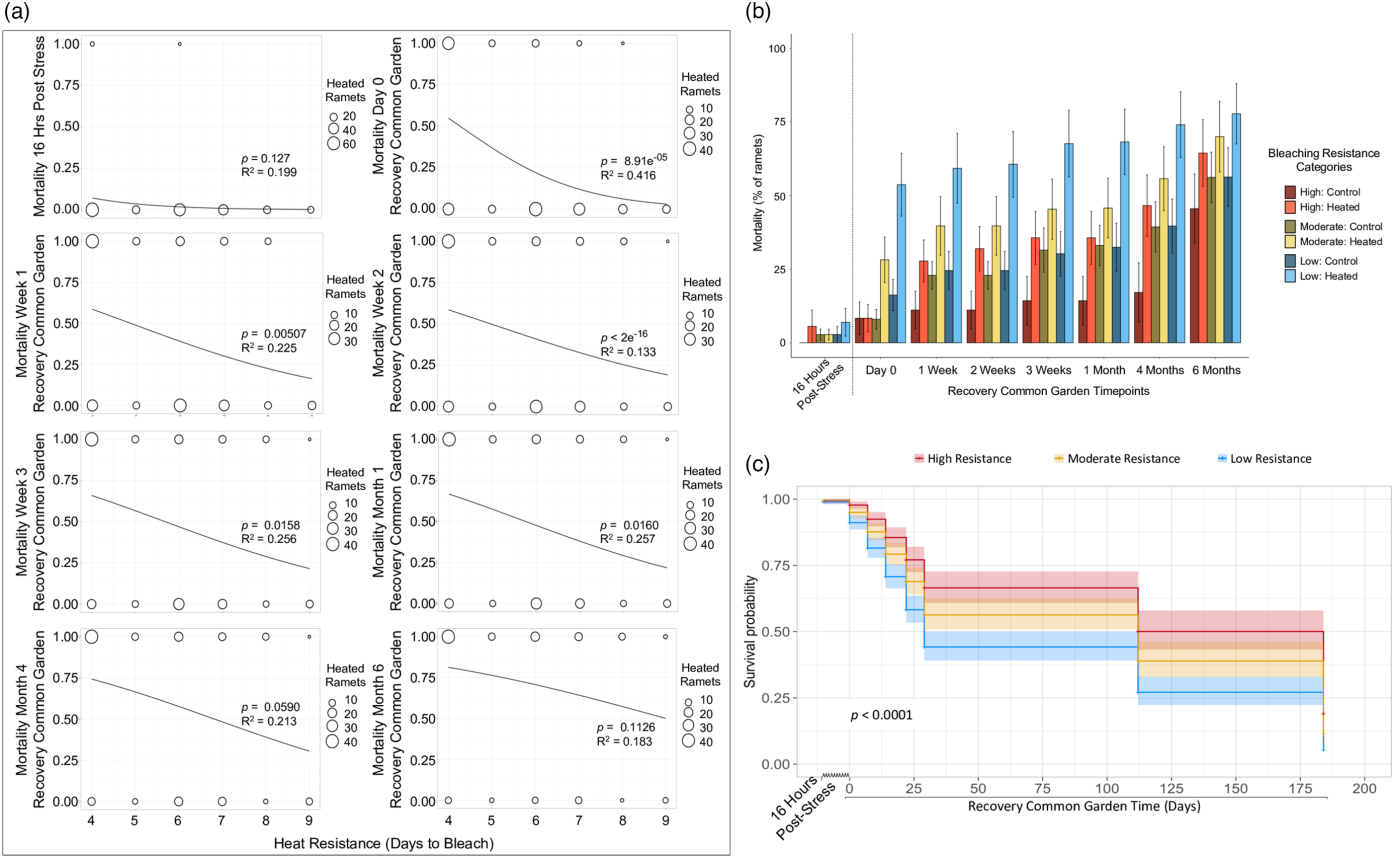
Mortality during the recovery common garden experiment, based on heat resistance. (a) Binomial plot of alive (value = 0) and dead (value = 1) heat‐stressed ramets at all postheat stress timepoints, in which heat resistance is grouped by number of days to bleach in the heat stress experiment (4–9 days) and circle sizes represent ramet sample size per group. Summarized statistical results from binomial linear mixed effects models evaluating the relationship between heat resistance and mortality are provided for each timepoint (Appendix S2). (b) Barplots showing the average (±*SE*) percentage of mortality across all heat‐stressed and control ramets, at the 16 h postheat stress timepoint (to the left of the black dotted line) and all available recovery common garden timepoints, grouped by broad heat resistance categories: Low (4 days to bleach), moderate (5–6 days), and high (7–9 days). (c) Heated ramet survival curves for the three broad heat resistance categories, beginning at the 16 h postheat stress timepoint and then through the common garden recovery experiment. The following timepoints are included: 16 h postheat stress, and common garden Day 0, Day 7 (Week 1), Day 14 (Week 2), Day 22 (Week 3), Day 29 (Month 1), Day 112 (Month 4), and Day 184 (Month 6). Survival probabilities were generated for each heat resistance category, based on the Cox proportional hazards model (Appendix S2).

While there was relatively little mortality 16 h after heat stress, 41% of all heated ramets died within 1 week after common garden deployment, compared to 20% mortality in controls. Further, 87% of all observed heated ramet mortality during the recovery experiment occurred within that first week (Figure 3, see Appendix S1). Heated ramet mortality continued to increase throughout the 6‐month recovery common garden experiment. Although mortality was greater in heated samples than controls, there was high mortality in controls at late common garden timepoints (Figure 3b). This finding suggested there may have been some additional stress on samples during transport to the reef common garden, there may be high natural turnover of corals in this system, or other biotic and abiotic factors such as disease and microclimate effects could have impacted mortality.

High‐resistant corals sustained less mortality than low‐ and moderate‐resistant corals during all recovery common garden timepoints (Figure 3a,b). Genets in the broader high‐resistance category had relatively low mortality after 1 week in the recovery common garden (28 ± 7%) compared to the moderate (40 ± 10%) and low (59 ± 12%) resistance categories (Figure 3b). Mortality throughout the early recovery period significantly decreased with heat resistance, though little of the variation was explained (recovery common garden Day 0 to Month 1 binomial mixed effects models *p* < 0.01, marginal *R* ^2^ ranged from 0.133 to 0.416, Figure 3a, Appendix S2). Mortality did not significantly decrease with heat resistance at the Month 4 and Month 6 recovery common garden timepoints (Figure 3a, Appendix S2). High‐resistance genets had a higher likelihood of survival over the 6‐month recovery period compared to other resistance categories (high vs. low Cox *p* = 1.4 e^−10^ and high vs. moderate Cox *p* = 0.0072, Figure 3c, Appendix S2), and genotype alone did not significantly impact ramet survival (Cox *p* = 0.53, Appendix S2). All resistance categories' survival probability dropped below 0.25 by recovery common garden Day 184 (Month 6) (Figure 3c).

Although we found high consistency in intra‐colony ramet mortality during heat stress, there was higher variability in mortality of intra‐colony ramets during recovery (Figure S2). No genets experienced mortality of all their heated ramets by the same timepoint. Ten genets had cumulative mortality of all ramets within 6 months of recovery—two high, three moderate, and five low‐resistant. Only two genets had total survival of all ramets throughout the recovery period—one low and one high resistant (Appendix S1). Average intra‐colony ramet mortality was 30 ± 5.8% on recovery Day 0, 50 ± 6.2% by recovery Week 3, and 71 ± 6.3% after 6 months (Figure S2 and see Appendix S1). This suggested there was some selective mortality within genotypes during recovery.

### 3.3 Chlorophyll concentration recovery based on heat resistance categories

**FIGURE 4:**
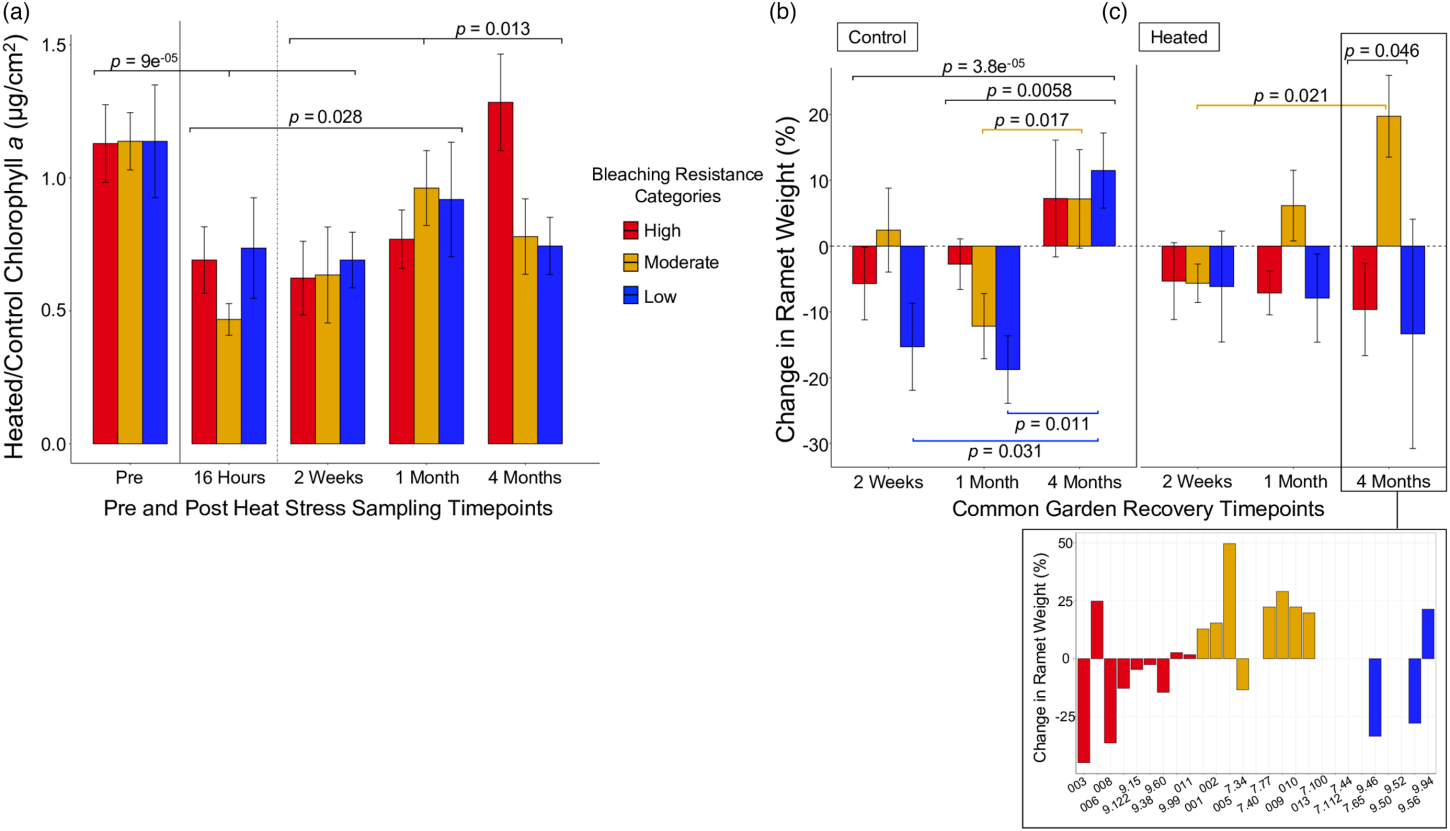
Chlorophyll *a* content and skeletal size change through recovery. Per genet, one available control and heat‐stressed ramet each were sacrificed at each timepoint. (a) Average (±*SE*) chlorophyll *a* (heated/control Chl *a* concentration, μg cm^−2^) of heat‐stressed versus control ramets categorized by heat resistance category—Prestress timepoint consisted of two nonstressed ramets per genet prior to the heat stress experiment. The solid black line divides pre‐ and postheat stress. The dotted black line divides 16 h postheat stress, which was prior to recovery common garden deployment, and the other recovery timepoints that occurred in the reef common garden. (b) Control and (c) heat‐stressed ramet average (±*SE*) buoyant weight change by heat resistance category, represented as percent change from the 16 h postheat stress weight. (c insert) Breakdown of skeletal weight change at the 4‐month recovery timepoint per genet. Kruskal–Wallis and post hoc pairwise Wilcox tests were used to evaluate the relationship between chlorophyll and heat resistance and between skeletal weight change and heat resistance throughout recovery. Shown are significant relationships between timepoints in black (Chl *a* and weight) and within resistance groups colored by category (weight) (see Appendix S2 for additional results).

We measured chlorophyll *a* concentration (Chl *a*) at preheat stress and poststress timepoints (after 16 h, and then after 2 weeks, 1 month, and 4 months in the recovery common garden) to quantify colony baseline pigmentation level, bleaching, and bleaching recovery. There were slight differences in baseline Chl *a* across different heat resistance categories, as has been shown previously (Cornwell et al., 2021), though these differences were not significant in our experiments (see Appendix S2). Average heat‐stressed ramet Chl *a* concentration was 37% lower 16 h postheat stress compared to controls and there were no significant differences in Chl *a* between heat resistance categories (see Appendix S2), confirming that genets experienced similar bleaching levels in response to different amounts of heat stress time. Among all heat‐stressed ramets, we found a significant decrease in chlorophyll *a* concentration 16 h poststress and 2 weeks into recovery compared to nonstressed controls (Kruskal–Wallis and post hoc Wilcox test, *χ* ^2^ = 27.323, *df* = 4, pre‐ vs. 16 h poststress and prestress vs. Week 2 recovery *p* = 9 e^−05^), but not at the 1 and 4‐month recovery timepoints (Figure 4a). This suggested heated ramets recovered pigmentation within 1 month of recovery. Chl *a* levels were not significantly different between resistance categories within any of our pre‐ or postheat stress timepoints (Figure 4a, see Appendix S2).

### 3.4 Skeletal growth trajectories following heat stress in recovering corals

We measured skeletal weight change in control and heat‐stressed ramets throughout recovery. Significant skeletal growth was not recorded in controls until 4 months postcommon garden deployment (Kruskal–Wallis and post hoc Wilcox test, *χ* ^2^ = 17.022, *df* = 2, Week 2 vs. Month 4 *p* = 0.0058 and Month 1 vs. Month 4 *p* = 3.8 e^−05^), at which point ramets grew on average 8.9 ± 4.0% compared to precommon garden deployment weight. Growth differences were insignificant between the controls of the three heat resistance categories (Figure 4b, see Appendix S2). This suggested that heat resistance capability was not strongly predictive of skeletal growth rate for nonheat‐stressed genets. Heat‐stressed ramets also exhibited little to no skeletal growth within the first month of the recovery period (Figure 4c, Appendix S2). However, differential skeletal growth 4 months into bleaching recovery was substantial based on heat resistance category. On average, low‐ and high heat‐resistant ramets did not grow within 4 months postbleaching (respectively, −13.3 ± 17.4% and −11.1 ± 7.0% compared to their starting weights). By contrast, the controls for these groups averaged 11.5 ± 5.7% (low resistance) and 7.2 ± 8.9% (high resistance) growth (Figure 4b,c). A different pattern was evident for the heat‐stressed, moderate‐resistance ramets, which on average grew by 19.7 ± 6.2% compared to their initial weight after 4 months of recovery. We found a significant difference in skeletal weight changes between the moderate and high‐resistance categories but not between the moderate and low‐resistance categories after 4 months of recovery (Kruskal–Wallis and post hoc Wilcox test, *χ* ^2^ = 6.502, *df* = 2, high vs. moderate *p* = 0.046 and moderate vs. low *p* = 0.200, Figure 4c), due in part to the especially high variation observed in the three remaining low‐resistance genets (Figure 4c). The moderate‐resistance heat‐stressed corals also recorded skeletal growth that was nearly three times greater than their nonstressed controls (which grew on average by 7.2 ± 7.5%) (Figure 4b,c), though this difference was not significant (Kruskal–Wallis, *χ* ^2^ = 1.5882, *df* = 1, *p* = 0.2076).

## 4 DISCUSSION

In this study, we collected *A. hyacinthus* genets with variable heat resistance capacity and exposed these individuals to different amounts of heat exposure time, to induce similar levels of bleaching. We then investigated heat stress recovery trajectories of corals grouped into three heat stress resistance categories based on the number of days to bleach: high, moderate, and low resistance. Chlorophyll *a* concentration rebounded within 1 month of heat stress recovery in corals regardless of initial heat resistance. High‐resistance corals experienced less mortality at all recovery timepoints, though these corals experienced increased mortality between 4–6 months of recovery. Higher‐resistant corals exhibited significantly less mortality throughout the first month of recovery but not after 4 and 6 months of recovery. Moderate‐resistance corals had significantly greater skeletal growth within 4 months of recovery compared to high‐resistance corals, and low‐ and high‐resistance corals on average did not exhibit skeletal growth within the 4‐month recovery period.

### 4.1 Heat resistance variation across different heat stress methods

In our experimental system, most genets bleached between 4 and 9 days, and a smaller subset died during heat stress (1–5 days) (see Appendix S1). Intraspecific heat resistance variability has been shown in many reef‐building corals around the world (e.g., Cornwell et al., 2021; Humanes et al., 2022; Muller et al., 2018; Sakai et al., 2019; Shaw et al., 2016; Tilstra et al., 2017; Walker et al., 2022). Twelve genets overlapped with another study on heat resistance variation, in which these higher resistant genets also exhibited higher symbiont retention during heat stress in Cornwell et al. (2021). Genets performed similarly across sampling years (2018 vs. 2019) and different heat stress methodologies (standardized 2‐day heat stress vs. variable and longer‐term heat stress assays), suggesting that intraspecific heat resistance variation can be consistently revealed across experiments. It has been previously shown that short‐ and long‐term heat stress assays can identify similar patterns of intraspecific variation within coral populations (Voolstra et al., 2020).

### 4.2 Intra‐colony variation during heat stress versus heat recovery

Our heat stress experimental system was designed to induce moderate bleaching in corals of variable heat resistance levels, initially determined by a visual bleaching score metric (Cornwell et al., 2021; Walker et al., 2022) and confirmed with chlorophyll *a* extraction. Most genets had all their ramets (*n* = 9 per genet) experience moderate bleaching (63% out of 27 genets) or mortality (83% out of 12 genets) on the same day of the heat stress experiment. The minimal intra‐colony variation observed during the heat stress experiment may have been driven by differences in symbiont populations (Garren et al., 2006; Kemp et al., 2014; Rowan et al., 1997; Ulstrup & van Oppen, 2003), or microbiome communities (Fifer et al., 2022; Marcelino et al., 2018). In contrast, we observed relatively high intra‐colony variability during the recovery period. Only 12 genets had either complete survival (*n* = 2) or mortality (*n* = 10) of all heat‐stressed ramets during the 6‐month recovery period, and the other 15 genets (56%) experienced mortality in some heated ramets throughout recovery. Similar to partial mortality of a coral colony following a bleaching event, intra‐colony mortality could have occurred due to factors such as predation, breakage, and disease when ramets were outplanted and left exposed to a natural reef environment during recovery (Lohr et al., 2020). Observed intra‐colony variation could also reflect phenotypic plasticity and selection associated with transitioning to the common garden reef environment (Drury et al., 2017; Lohr et al., 2020). The holobiont stress response in the hours to days following heat stress is tightly regulated (Palumbi et al., 2014; Seneca & Palumbi, 2015; Thomas et al., 2018; Traylor‐Knowles et al., 2017), with high intra‐colony consistency observed across different durations and levels of stress intensity (Cziesielski et al., 2019; Dixon et al., 2020; Morikawa & Palumbi, 2019). However, longer‐term stress repair, recovery, and reestablishment of homeostasis may have greater stochasticity as corals manage survival in variable and dynamic marine environments.

### 4.3 Correlations between heat resistance and mortality

Mortality during the recovery period varied based on relative heat resistance, in which higher heat‐resistant genets (i.e., more days under heat stress to bleach) consistently experienced less mortality than lower‐resistant genets. Mortality across all genets predominantly occurred within the first month of recovery. Significant mortality can occur within the first week postheat stress in experimental systems (Walker et al., 2022) and up to 1–2 months afterward in experimental and natural settings (Matsuda et al., 2020; Walker et al., 2022). A difference in mortality between heated and control ramets of higher heat‐resistant genets did not emerge until 1 week following heat stress. There was also an increase in mortality of heated ramets of higher heat‐resistant genets between 4‐ and 6‐month postbleaching, which may have signaled delayed heat stress impacts in these genets. Damage sustained during experimental heat stress may have continued to interfere with survivability during recovery (Grottoli et al., 2014; Schoepf, Grottoli, et al., 2015; Schoepf, Stat, et al., 2015; Thomas et al., 2019; Thomas & Palumbi, 2017). Higher heat‐resistant genets maintained high pigmentation for 3–5 days longer than lower‐resistant genets and may have been more vulnerable after heat stress due to higher energy demands needed to sustain a longer‐term heat stress response (Maor‐Landaw et al., 2014; Maor‐Landaw & Levy, 2016; Williams et al., 2021) or exposed to higher levels of symbiont stress byproducts such as radical oxygen species (Buerger et al., 2020; Cziesielski et al., 2018; Downs et al., 2002; Meron et al., 2019).

### 4.4 Chlorophyll recovery

Heated ramets had significantly lower chlorophyll *a* concentration 16 h and 2 weeks after heat stress compared to controls but not after 1 and 4 months, suggesting that pigmentation rebound occurred within 1 month of recovery. This is consistent with previous studies, in which other bleaching‐sensitive species have also fully recovered chlorophyll concentrations within 4 months of recovery (Rodrigues & Grottoli, 2007; Schoepf, Grottoli, et al., 2015; Schoepf, Stat, et al., 2015; Wall et al., 2019). There were no significant differences in chlorophyll a concentration between heat resistance categories, which showed similar paces of pigmentation rebound across observed recovery timepoints. However, significant differences between heat resistance categories may have been obscured in part by high variation of chlorophyll measurements within categories and genets. For example, at the preheat stress timepoint, we tested two ramets per genet, which showed an average 31 ± 7% difference in Chl *a* (see Appendix S1). This may be improved upon in future studies by increasing the sample size or including additional ramets per measurement.

Previously, a relationship between symbiont load (i.e., symbiont counts preceding heat stress) and heat resistance was shown in *A. hyacinthus* (Cornwell et al., 2021) and *Pocillopora damicornis* (Cunning & Baker, 2013). In our experiments, there were slight but statistically insignificant differences in chlorophyll *a* load prior to heat stress, in which high‐ and moderate‐resistance genets had less starting pigmentation and yet took 1–5 days longer than low‐resistance genets to bleach. Overall, this study did not find strong evidence for the symbiont load versus heat resistance tradeoff, although further research may confirm potential impacts of this putative tradeoff.

### 4.5 Growth tradeoffs associated with high heat resistance

There was comparable common garden growth in controls across resistance categories. However, among heat‐stressed ramets, only those from moderately resistant genets grew on average during recovery. Coral skeletal growth has been used as a proxy for health (Wright et al., 2019), and it is possible that moderate‐resistance genets' skeletal growth outpaced high‐ and low‐resistance genets due to higher fitness or differences in energy reserves or allocation during heat stress recovery (Grottoli et al., 2006, 2014; Rodrigues & Grottoli, 2007). Heterotrophic plasticity may have also contributed to skeletal growth patterns, in which moderate‐resistance genets could have preferentially increased heterotrophy early during recovery when symbiont concentrations were low (Grottoli et al., 2006; Levas et al., 2016), compared to genets in other resistance categories. This experimental design did not include supplemental feeding during the heat stress experiment, and we did not measure heterotrophic capacity (e.g., feeding rates and heterotrophic vs. photoautotrophic derived carbon) of recovering corals on the reef. Accounting for heterotrophic plasticity in future studies may contribute to further illuminating the observed differences during recovery. Additionally, there may be calcification tradeoffs from symbiont switching following bleaching (Bay et al., 2016; Jones & Berkelmans, 2010; Little et al., 2004; Thomas et al., 2019), which may have played a role in skeletal growth differences observed in this system. Therefore, investigating symbiont type pre‐ and postheat stress could further explain skeletal growth patterns.

There may be longer‐term physiological tradeoffs associated with expelling symbionts quickly or retaining a high concentration of symbionts longer. Other marine systems have suggested there are moderate stress resistance “sweet spots” that are linked to longer‐term stress recovery. For example, long‐term resilience among certain seagrass species has been linked to moderate physiological resistance coupled with rapid and high recruitment potential for effective transgenerational recovery (Kilminster et al., 2015; McKenzie & Yoshida, 2020). Moderately resistant heat‐stressed ramets also grew on average three times more than their controls, although this growth was not statistically different. Future research into whether heat stress may enhance skeletal growth in moderate‐resistance genets may reveal important fitness implications. It has been demonstrated in other organisms that short‐term, sublethal stress may encourage preferential investment of energy into life processes such as growth (e.g., Leung & McAfee, 2020). It has also been widely shown in woody plant agricultural systems that pruning—the act of removing dead or overgrown branches or stems—can stimulate healthy fruit growth (Albarracín et al., 2017; Ashraf & Ashraf, 2014; Mika, 1986). Here, moderate‐resistance genets were able to maintain, and potentially surpass, normal skeletal growth rates following heat stress.

### 4.6 Incorporating resistance and recovery tradeoffs into management programs informed by resilience

This study highlights the importance of investigating intraspecific variation and resilience tradeoffs in reef‐building corals. Intraspecific variation is the building block material for species acclimation and adaptation (Di Santo, 2016; Stitt et al., 2013), and intentional incorporation of variation into management may lead to more successful rehabilitation programs (Bremner, 2008). Thermal resilience is a complex and dynamic trait that can in part be influenced by genetics (Barshis et al., 2013), intensity, and frequency of stress‐inducing events (Hughes et al., 2017), and microbiome and species interactions (Parkinson & Baums, 2014). Selecting only one aspect of resilience (e.g., high heat resistance) may result in lower performance in other vital areas (e.g., lower fitness during heat recovery) and lead to longer‐term consequences, such as lower population diversity and fitness (Kristensen et al., 2006).

Here, although high heat resistance genets have lower mortality during recovery and corals from all heat resistance categories recover chlorophyll *a* levels within 1 month of recovery, we find that moderate‐resistance genets have the highest skeletal growth and may exhibit higher fitness during recovery. Both theoretical (Walsworth et al., 2019) and empirical studies (Barshis et al., 2013; Hughes et al., 2017; Morikawa & Palumbi, 2019; Schoepf et al., 2019; Thomas et al., 2018; West & Salm, 2003) show that high heat‐resistant corals can survive bleaching to generate a coral population with higher overall resistance to ocean warming. However, high resistant corals may already be nearing their limits in warming environments and unable to continue effectively adjusting to increased ocean warming (Voolstra et al., 2021). Incorporating intraspecific variation of resilience as a complex trait that involves resistance and recovery may be necessary for management programs to succeed in increasing coral population resilience in the face of climate change. Otherwise, major efforts in assisted evolution, gene flow, or restoration may have unintended consequences that limit their beneficial effects.

### 4.7 Future directions and concluding remarks

We have developed an acute‐to‐moderate duration (Grottoli et al., 2021) heat stress experimental system that reliably reveals intraspecific variation in heat resistance. Further, we demonstrate that heat resistance variation is linked to differential mortality and skeletal growth during heat recovery. Based on our data, moderate‐resistance corals may exhibit faster heat recovery compared to high‐ and low‐resistance corals, as suggested by relatively high skeletal growth compared to other corals. If corroborated in follow‐up experiments with other species and on other reefs, these results would strongly suggest that heat resistance is not an adequate measure of future coral resilience to bleaching and that recovery is a second key feature. As a result, this finding has the possibility to dramatically change approaches to maintenance, recovery and enhancement of coral resilience using a wide range of possible interventions. Future research on links between resistance and recovery should also investigate genetics and genomics, host‐symbiont interactions, and further physiological measures such as host energy reserves and heterotrophic capacity.

## CONFLICT OF INTEREST

On behalf of all authors, the corresponding author states that there is no conflict of interest.

## Supporting information


Figure S1

Figure S2

Appendix S1

Appendix S2

## Data Availability

Raw data and underlying results are included as supplemental metadata files.
