# Supplementary figures and images for: Coral bleaching resistance variation is linked to differential mortality and skeletal growth during recovery
Source: Evol Appl. 2022 Nov 7;16(2):504–17. doi: 10.1111/eva.13500 (PMC9923480; doi:10.1111/eva.13500)

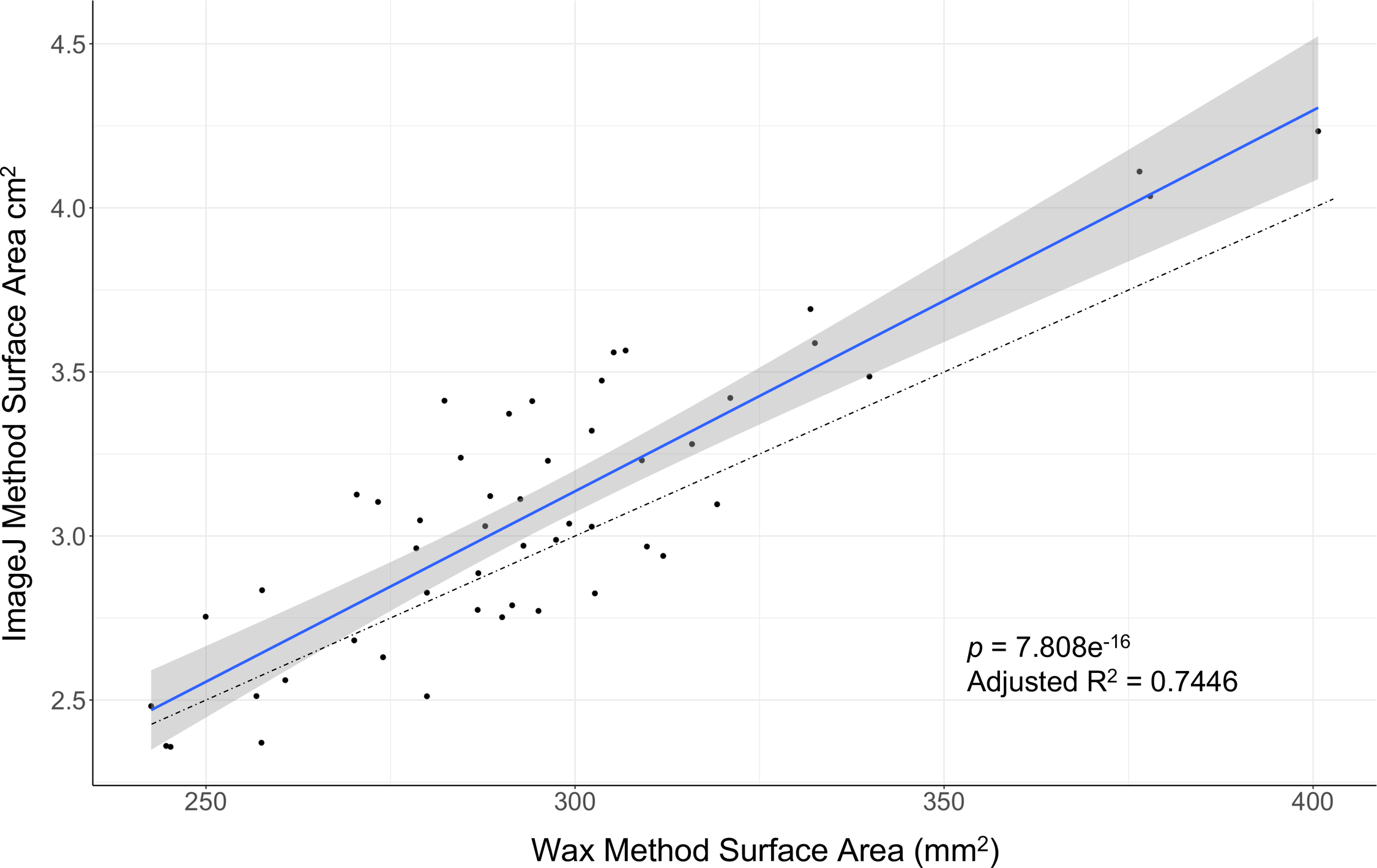

Supplement: Supplementary file 1 — Figure S1 [file EVA-16-504-s004.tif]

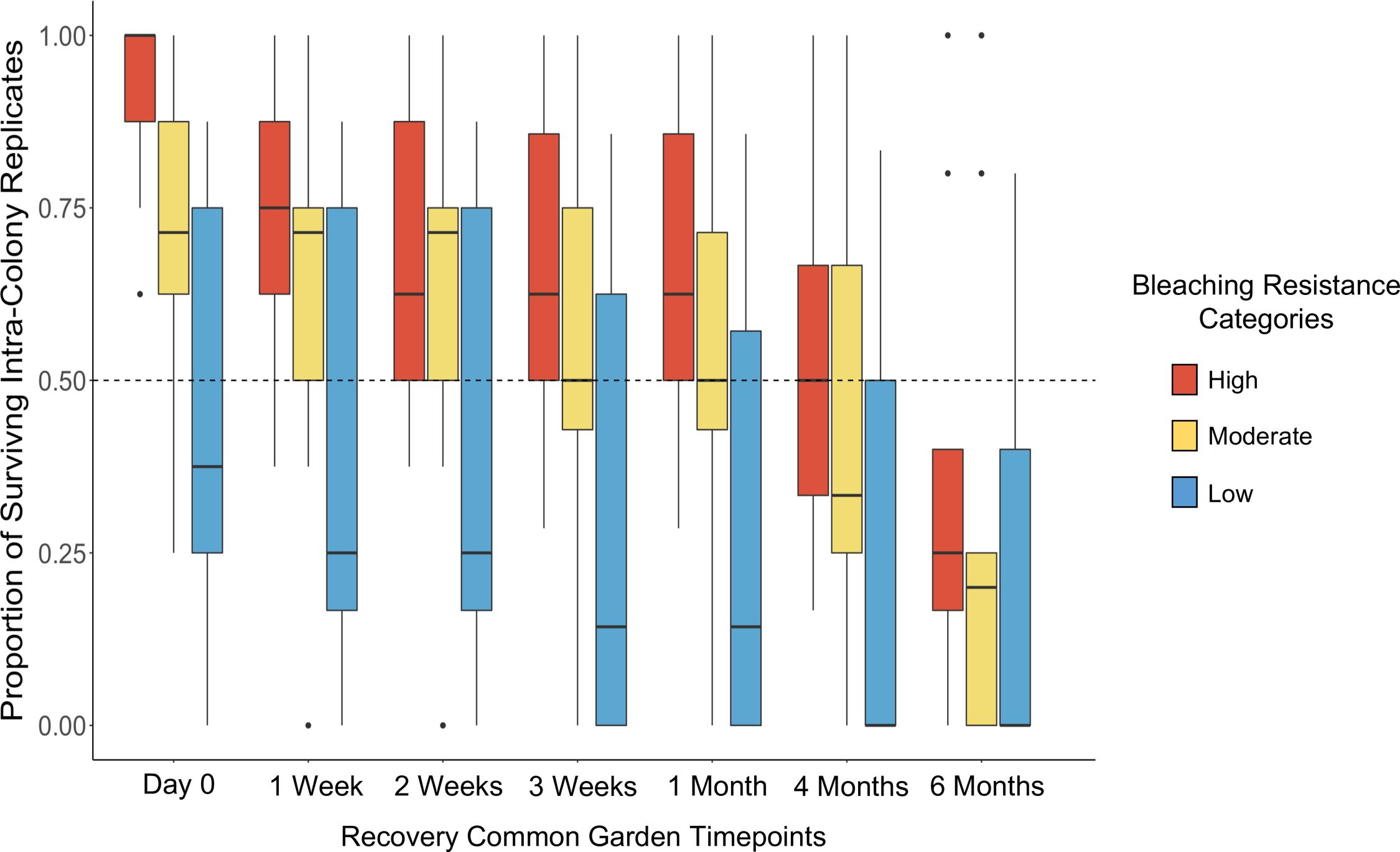

Supplement: Supplementary file 2 — Figure S2 [file EVA-16-504-s001.tif]
